# Thermomechanical Parameters Modelling of Spring Force Elements Made of Shape Memory Alloys

**DOI:** 10.3390/ma18133055

**Published:** 2025-06-27

**Authors:** Olga Łastowska, Vitaliy Polishchuk, Andrii Poznanskyi

**Affiliations:** 1 Faculty of Marine Engineering, Gdynia Maritime University, 81-87 Morska St., 81-225 Gdynia, Poland; 2 Department of Engineering Mechanics and Machine-Building Technologies, Admiral Makarov National University of Shipbuilding, 9 Heroiv Ukrainy Ave, 54025 Mykolaiv, Ukraine; vitpolishchuk@gmail.com (V.P.); andrii.poznanskyi@nuos.edu.ua (A.P.)

**Keywords:** shape memory alloys, shape memory effect, thermomechanical modelling, phase transformation kinetics, thermal control devices, thermosensitive element, actuating mechanism

## Abstract

This study presents a phenomenological model for predicting the thermomechanical behaviour of spring-type actuators made of shape memory alloys (SMAs). The model incorporates the kinetics of martensite–austenite phase transitions as a function of temperature and applied stress. The primary innovation is the inclusion of a scalar internal variable that represents the evolution of the phase transformation within a phenomenological macroscopic model. This approach enables the deformation–force–temperature behaviour of SMA-based spring elements under cyclic loading to be accurately described. A set of constitutive equations was derived to describe reversible and residual strains, along with transformation start and finish conditions. Model parameters were calibrated using experimental data from VSP-1 and TN-1K SMA springs that were subjected to thermal cycling. The validation results show a high correlation between the theoretical predictions and the experimental data, with deviation margins of less than 6.5%. The model was then applied to designing and analysing thermosensitive actuator mechanisms for temperature control systems. This yielded accurate deformation–force characteristics, demonstrating low inertia and high repeatability. This approach enables the efficient prediction and improvement of the performance of SMA-based spring elements in actuators, making it relevant for adaptive systems in marine and aerospace applications.

## 1. Introduction

Shape memory alloys (SMAs) are a class of functional materials that exhibit unique properties, such as the shape memory effect (SME) and superelasticity. These properties enable the alloys to recover their original shape when heated or unloaded following deformation. These behaviours result from reversible martensitic transformations between austenite and martensite phases, which are highly sensitive to temperature, stress, and strain rates. NiTi-based SMAs are widely utilised due to their excellent mechanical properties, corrosion resistance, and fatigue life.

Recent research has increasingly focused on integrating SMAs into adaptive and energy-efficient systems, including actuators, sensors, and energy harvesters. Sophianopoulos and Ntina [1] demonstrated the effectiveness of incorporating X-braced NiTi SMA elements into multi-story truss moment frames, significantly enhancing seismic performance by reducing residual drifts and ensuring self-centering capability. Goryczka et al. [2] explored phase composition evolution in Ni-rich NiTi alloys subjected to high-energy ball milling, highlighting how amorphous–nanocrystalline mixtures dominate the resulting structure and how increasing nickel content modulates the relative phase fractions. Stolbova and Stolbov [3] introduced a coupled thermomagnetic model of a bi-layer cantilever composed of NiTi and magnetoactive elastomer, establishing the feasibility of dual-stimulus control in microactuation.

Jo [4] proposed a temperature-tunable photonic crystal sensor using SMAs, demonstrating the real-time shift of defect-band frequencies via thermally induced modulus changes, thus enabling adaptive ultrasonic sensing. Milosavljevic et al. [5] analyzed Cu-Al-Mn SMA thin sheets and showed that thermomechanical treatments such as cold rolling and aging influence transformation temperatures and manganese retention, thereby tuning functional properties. Dmitrievskiy et al. [6] assessed nanostructured TiNi’s tribological properties, confirming that severe plastic deformation significantly enhances hardness and wear resistance without compromising superelastic performance.

Yotov et al. [7] developed a thermal energy harvester using SMA-induced vibrations to actuate piezoelectric cantilevers, reporting stable output voltage generation and highlighting the potential for miniaturisation and autonomous power supply in sensor systems. Tymoshchuk et al. [8] applied machine learning to classify SMA loading frequencies based on stress–strain data, identifying multilayer perceptrons as highly accurate and emphasising the role of data-driven modelling in fatigue diagnostics. Bizzarri et al. [9] showed that strain rate substantially affects the elastocaloric performance of NiTi sheets, with higher rates increasing transformation stress and energy dissipation, thereby guiding the optimisation of SMA-based cooling devices. Extending SMA exploration to Fe-based systems, Matcovschi et al. [10] investigated SMAs produced by powder metallurgy and mechanical alloying. 

The fracture and fatigue behaviour of NiTi shape memory alloys (SMAs) is strongly influenced by their microstructure, processing route, and thermomechanical treatment [11]. Recent studies emphasize the role of stress-induced martensitic transformation near crack tips, as well as complex fracture mechanisms driven by reversible phase changes and transformation-induced plasticity [12]. Understanding these effects is crucial for the reliable use of SMAs in advanced applications beyond biomedical devices, particularly in actuators and energy-absorbing systems [13]. Together, these studies highlight the evolving capabilities of SMAs, including novel processing routes, functional modelling, and integration into adaptive systems for mechanical, thermal, and electronic applications.

Unlike many existing models which rely on complex crystallographic parameters or are limited to discrete phase states, our model integrates phase transformation kinetics and deformation response into a single system of constitutive equations, applicable to nonlinear spring-type actuators.

The aim of this study is to develop a phenomenological model describing the thermomechanical behaviour of spring-type elements with a shape memory effect, intended for use in cyclic actuators of various technical systems. The model accounts for the influence of temperature on the shape of compression diagrams and their evolution under thermomechanical loading.

## 2. Methods

For the realisation of reciprocating motion in actuators of thermal control devices and cyclic-action drives of various technical systems, thermomechanical drive schemes utilising thermosensitive elements in the form of spiral springs with a shape memory effect are considered to be among the most promising. Spring-type thermosensitive elements offer an optimal combination of displacement amplitude and generated force, ease of adjustment of these parameters, and an actuation temperature range. They also offer high cyclic durability, structural simplicity, and ease of integration within the assembly unit [14,15,16].

An analysis of existing calculation methods and thermomechanical models of shape memory alloys undergoing phase transitions shows that phenomenological or macroscopic models are preferable to metallurgical or micromechanical models for calculating the parameters of these alloys and devices under complex thermal and deformation–force conditions [15,17,18,19,20,21,22].

When solving practical problems, it is advisable to consider the phase transition as a process that occurs over a finite time interval. This approach enables the mechanical characteristics of force elements made of shape memory alloys to be obtained, which are necessary for developing thermomechanical drives in the form of time-dependent force responses generated by the elements [23,24].

From this perspective, the following approaches to the modelling and design of thermosensitive elements for control and power devices appear promising. In the comprehensive studies [24,25], the strain increment *d*ε of a thermosensitive element is considered as resulting from the elastic component *d*ε*^e^* (taking into account the deformation *d*ε*^eT^* caused by the temperature dependence of elastic constants), as well as deformations due to the volumetric transformation effect *d*ε*^Q^*, transformation-induced plasticity *d*ε*^pl^*, shape memory effect *d*ε*^sm^*, conventional dislocation plasticity *d*ε*^p^*, and thermal expansion *d*ε^α^. An important aspect lies in the introduction of the following relationships:

*d*ε*^pl^* = *f*_1_(*d*Φ) and *d*ε*^sm^* = *f*_2_(*d*Φ) (1)

according to which the strain rates *d*ε*^pl^*/*dt* and *d*ε*^sm^*/*dt* are determined by the rate of the phase transformation *d*Φ/*dt*, which in turn depends on the rate of temperature change (*d*Φ/*dt* ~ *dT*/*dt*) (where Φ is normalised per unit amount of the forming phase). The proposed approach allows for a fairly accurate description of the elastic–plastic behaviour of thermosensitive elements with a shape memory effect (SME) within the temperature range of phase transformation. However, it is based on the crystallographic interpretation of the SME phenomenon, which necessitates the use of a large number of microscopic state parameters of the thermosensitive element material (such as tensors of oriented microstresses, coefficients of thermal expansion, coefficients of reversible strain hardening, loading and hardening parameters, parameters related to the mechanism of phase transformation and material properties, crystallographic distortion of transformation, components associated with accommodation processes accompanying the phase transition, anisotropy of thermal expansion coefficients, and coefficients accounting for the contributions of the strains ε*^p^*, ε*^pl^*, and ε*^sm^* to the generation of microstresses and the share of the corresponding strain recovery, etc.). This leads to significant difficulties in obtaining the necessary initial data and substantially complicates the calculations of actuators based on shape memory alloys, often resulting in considerable discrepancies between theoretical predictions and experimental results due to the impossibility of accurately determining the initial parameters.

Similar difficulties arise when using phenomenological models based on thermoplasticity theory to calculate the behaviour of thermosensitive elements with a shape memory effect. This theory is founded on the local form of the second law of thermodynamics in the form of the Clausius–Duhem inequality [26,27]. It should be noted that, due to the complexity of the mathematical description of the phase transformation in the material of the thermosensitive element, the theory proposes limiting the state variables within certain bounds, thereby restricting the validity of the equilibrium equations to the regions where the phase transformation occurs. In this model, temperature *T* and the components of the stress tensor σ are considered as independent variables. At the same time, *T* must not significantly deviate from the phase transformation temperatures, and the stress σ must be limited to values corresponding to reversible changes in the material state, remaining below the dislocation yield strength of the shape memory alloy.

Graph-analytical calculation methods [18] and a number of mathematical models [19,25] describing the thermomechanical behaviour of actuators based on shape memory alloys operate with macroscopic parameters (temperature, stress, strain), which ensures their simplicity and satisfactory accuracy. However, these models have a significant draw-back—they describe discrete states of the shape memory alloy in the initial and martensitic phases. This approach is sufficient for calculating thermosensitive elements of discrete devices (mainly of two-position type), but it is not suitable for calculating the operational parameters of adaptive and thermosensitive elements in devices and systems requiring continuous regulation.

A common drawback of the models considered is that they describe “pure” types of stress states (tension–compression, torsion, bending), which are mainly applicable only to linear actuators. When designing nonlinear, particularly spring-type, thermosensitive elements based on shape memory alloys, it must be taken into account that the stress σ—strain ε curves of these alloys are nonlinear, and both the shear modulus and the elastic constant are not true constants. Therefore, general methods for calculating spiral springs are not applicable. Moreover, the curve σ = *f* (ε) changes depending on the material’s thermal or deformation history, and there is a lack of sufficiently complete data regarding the properties under torsion. As a result, the precise design of spring-type thermosensitive elements with specified properties is challenging.

The method of calculating spring-type thermosensitive elements using an apparent spring constant, and the graphical method of determining the load–deflection curve of SME spirals (with sequential measurement of values at different stages of the τ–γ curve, independently of the spring constant) are based on well-known methods of calculating spiral springs, with the introduction of stress correction factors [18,28]. These methods provide an approximate solution and are therefore only advisable for cases involving small deformations. Furthermore, these methods cannot model the behaviour of thermosensitive elements across the entire temperature range of the phase transformation and are only applicable to discrete-action devices.

In [23], thermomechanical models of thermosensitive elements made of shape memory alloys during phase transformation are considered phenomenologically, using continuum mechanics with an internal variable. However, this theory has only been developed for uniaxial stress states and requires certain refinements, particularly with regard to the dependence of elastic moduli, deformation characteristics, and material parameters on temperature and the degree of phase transformation completion.

In summary, the phase transformation process in thermosensitive materials with a shape memory effect can be well described by introducing internal variables that characterise the degree of transformation development [23,24,25]. The form of these variables (scalar, vector or tensor of various ranks) depends on the specific problem conditions. According to such a model, the development of the phase transformation process in continuum mechanics obeys an evolutionary constitutive equation, referred to in metallurgy as the phase transformation kinetics relation.

## 3. Model

In the process of thermomechanical martensitic transformation in the material of power elements, we will consider only two phases—martensite and “austenite”. Therefore, we introduce the volumetric fraction of martensite, denoted by the scalar internal variable ξ. Consequently, the volumetric fraction of the high-temperature phase will be equal to 1 − ξ.

Let us consider a body made of a shape memory alloy after stabilising thermomechanical cycling, which is in a state of thermoelastic martensitic transformation or reverse phase transition. The thermomechanical parameters of the material are determined by equations of the following form:

(2) $$\frac{d\sigma }{dt}=f\left(\frac{d\varepsilon }{dt},\frac{dT}{dt},\frac{d\xi }{dt}\right),\;\xi =\varphi \left(\sigma ,T\right),$$

where σ and ε denote the stress and strain tensors, respectively; *T* is the sample temperature; *t* is time.

The second Equation (2) describes the process (kinetics) of the phase transformation, the degree of which depends solely on the current values of the arguments. This reflects the fact that martensitic transformation is not a diffusion-controlled process. The presence of the stress tensor among the arguments indicates that the transformation depends not only on temperature but also on the applied stress.

It is important to note that the equations

0 = *φ*(*σ*, *T*), 1 = *φ*(*σ*, *T*) (3)

derived from the second Equation (2) define boundary surfaces in the stress–temperature space. The positions of the points (σ, *T*) on these surfaces correspond to the start and end of the phase transformation, respectively, while all points located between these surfaces correspond to the progression of the phase transformation process.

For a spring-type force element made of shape memory alloy, the system of Equation (2) can be expressed in the following form:

(4) $$\left\{\begin{array}{c} \frac{d\tau }{dt}=K\frac{d}{dt}(G{\left[\gamma -\gamma_{\mathrm{rev}}-\gamma_{\mathrm{res}}\right]}^{m});\\ \xi =1-e^{a_{M}b_{M}(M_{S}-T)+a_{M}\tau };\\ \xi =e^{a_{A}b_{A}(A_{S}-T)+a_{A}\tau }; \end{array}\right.$$

under the conditions

(5) $$\begin{array}{c} a_{M}b_{M}(M_{S}-T)+a_{M}\tau \leq 0;\\ a_{A}b_{A}(A_{S}-T)+a_{A}\tau \leq 0;\\ \gamma_{\mathrm{res}}+\gamma_{\mathrm{rev}}\leq \gamma \leq \gamma_{\mathrm{in}}, \end{array}$$

where the first equation of system (4) describes the relationship between deformation–force and temperature parameters of spring elements with SME within the temperature range of the phase transformation, while the second and third equations of system (4), describing the kinetics of the direct and reverse phase transformations, respectively, represent a natural generalisation of the kinetics of martensitic thermal transformation [9,10]. Here, parameter *τ*—maximum shear stresses in the material of the spring coil; *K* and *m*—material parameters determined based on experimental data; *a_M_*, *b_M_*, *a_A_*, *b_A_* are material parameters that, in the general case, depend on temperature; *G*—the shear modulus, which is a function of the internal variable ξ and *G* = *A*ξ + *B*; *M_S_* (*M_F_*) and *A_S_* (*A_F_*) are the characteristic temperatures of the start (end) of the forward and reverse martensitic transformation, respectively; γ*_rev_*—reversible shape memory strain (γ*_rev_* = *K*_1_ξ);

γ*_res_*—total residual strain accumulated after thermomechanical cycling; determined as a function of the number of thermal-cycles *N* and the strain γ_in_ induced to initiate the SME according to the formula: γ*_res_* = *u*_0_ (γ*_in_*) + *u*_1_ (γ*_in_*) exp [*Nu*_2_ (γ_in_)], where *u*_1_ (γ*_in_*) = *C_i_*γ*_in_* + *D_i_* and *C_i_*, *D_i_*—constants characterising the cyclic properties of the alloy [4].

The coefficients *A*, *B*, *K*_1_ are determined from the following boundary conditions:

if *T* < *M_F_*:
  (6) $$\xi =1,\;G=G_{\text{M}},\;\gamma_{\mathrm{rev}}=\gamma_{\mathrm{rev}}^{\max}$$
if *T* > *A_F_*:
ξ = 0, *G* = *G_A_*, γ*_rev_* = 0 (7)
  where *G_A_* and *G_M_*—are the shear moduli of the initial and martensitic phases, respectively.

Then, *A* = *G_M_* − *G_A_*; *B* = *G_A_*; $K_{1}=\gamma_{\mathrm{rev}}^{\max}$ and consequently,

(8) $$G(\xi )=(G_{M}-G_{A})\xi +G_{A}$$

(9) $$\gamma_{\mathrm{rev}}(\xi )=\gamma_{\mathrm{rev}}^{\max}\xi$$

The value $\gamma_{\mathrm{rev}}^{\max}$ of the maximum strain, realised due to reversible SME upon cooling the sample below the temperature *M_F_*, is determined as a function of the number of thermal-cycles *N* and the SME initiation strain γ_in_ (similarly to the residual strain):

(10) $$\gamma_{\mathrm{rev}}^{\max}=\nu_{0}(\gamma_{\mathrm{in}})+\nu_{1}(\gamma_{\mathrm{in}})\exp[N\nu_{2}(\gamma_{\mathrm{in}})]$$

where ν_i_ (γ_in_) = *c*_i_γ_in_ + *d*_i_; *c*_i_, *d*_i_ are constants that characterise the cyclic properties of the alloy [17].

When deforming the spring element, we only consider torsional stresses, meaning that the surfaces at the start and end of the phase transition degenerate into lines, and the equations for which can be determined from the second and third equations of the system (4). In deriving the equations for the phase transition end lines, the transition is considered complete when the relative fraction of the new phase reaches a value of 0.99.

For the martensitic transformation, we have the following equations:

– the start of the transformation
0 = 1 − exp [*a_M_b_M_*(*M_S_* − *T*) + *a_M_*τ] (11)
– the end of the transformation
0.99 = 1 − exp [*a_M_b_M_*(*M_S_* − *T*) + *a_M_*τ] (12)

It follows that

(13) $$\begin{array}{l} \tau =b_{M}\left(T-M_{S}\right);\\ \tau =-\frac{2\ln10}{a_{M}}+b_{M}\left(T-M_{S}\right). \end{array}$$

For the austenitic transformation, we have

– the start of the transformation
1 = exp [*a_A_b_A_*(*A*_S_ − *T*) + *a_A_*τ]; (14)
– the end of the transformation
0.01 = exp [*a_A_b_A_*(*A*_S_ − *T*) + *a_A_*τ]. (15)

It follows that

(16) $$\begin{array}{l} \tau =b_{A}(T-A_{S});\\ \tau =-\frac{2\ln10}{a_{A}}+b_{A}(T-A_{S}). \end{array}$$

It should be noted that the lines indicating the start of the phase transformation correspond to the equality condition in Equation (5). The region between the start and finish lines of the transformation can be referred to as the phase transformation band. The transformation completion lines intersect the abscissa axis at the points *M_F_* and *A_F_*, which can be determined from the second equations of systems (13) and (16) by setting *τ* = 0:

(17) $$\begin{array}{l} a_{M}b_{M}=\frac{2\ln10}{M_{F}-M_{S}};\\ a_{A}b_{A}=\frac{2\ln10}{A_{F}-A_{S}}, \end{array}$$

where the first equation corresponds to the martensitic transformation, while the second describes the reverse martensitic transformation.

**Figure 1:**
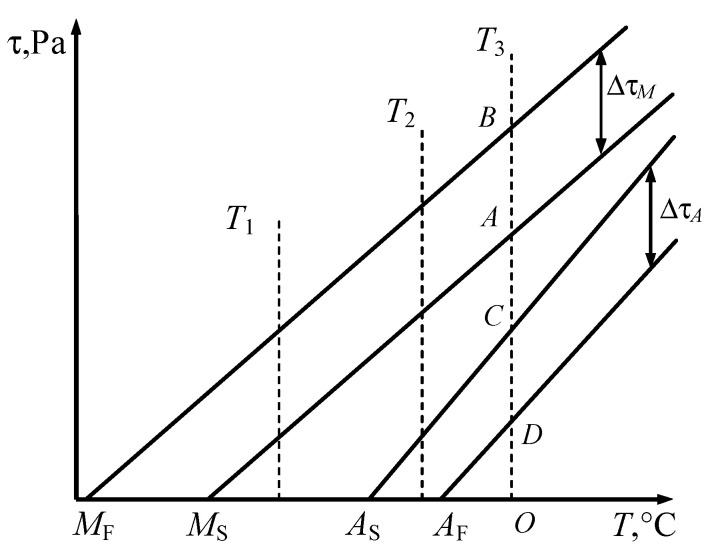
Boundaries and transformation bands of phase transitions.

Since *M_F_* < *M_S_* and *A_F_* > *A_S_*, the boundaries and transformation bands can be schematically represented as shown in Figure 1 in the stress–temperature plane.

The width of the phase transformation bands Δτ*_M_* and Δτ*_A_* along the ordinate axis is calculated using the equations of systems (13) and (16):

(18) $$\begin{array}{l} \Delta \tau_{M}=-\frac{2\ln10}{a_{M}};\\ \Delta \tau_{A}=\frac{2\ln10}{a_{A}}. \end{array}$$

Equation (18) shows that *a_M_* < 0 and *a_A_* > 0. From Equation (17), we obtain that both parameters *b_M_* and *b_A_* are positive. In this case, inequalities (5) simplify and take the following form:

(19) $$\begin{array}{l} \tau \geq b_{M}(T-M_{S});\\ \tau \leq b_{A}(T-A_{S}). \end{array}$$

It is also important to bear in mind that, when the material parameters are temperature independent, phase transitions are only possible under thermomechanical conditions of the following form (derived from inequalities (19)):

(20) $$\begin{array}{l} \frac{d\tau }{dt}-b_{M}\frac{dT}{dt}>0,\\ \frac{d\tau }{dt}-b_{A}\frac{dT}{dt}<0. \end{array}$$

Equations (17) and (18) enable the values of the material parameters that determine the kinetics of the phase transition to be calculated. If these parameters remain constant throughout the process, the phase transformation onset lines are straight. This case is described by the Clausius–Clapeyron relation [18,21].

It should be noted that when the width of the phase transformation bands Δτ*_M_* and Δτ*_A_* remains constant within the considered temperature range, the material parameters *a_M_*, *b_M_*, *a_A_*, and *b_A_* are assumed to be constant (the phase transformation lines are nearly linear). However, in many cases, the width of the martensitic or austenitic transformation band depends linearly on temperature:

(21) $$\begin{array}{l} \Delta \tau_{M}=a_{1}+a_{2}T,\\ \Delta \tau_{A}=b_{1}+b_{2}T, \end{array}$$

where *a_i_* and *b_i_* are material parameters.

In this case, from Equations (18) and (21), we obtain the following expressions for the coefficients *a_M_* and *a_A_*:

(22) $$\begin{array}{l} a_{M}=-\frac{2\ln10}{a_{1}+a_{2}T};\\ a_{A}=\frac{2\ln10}{b_{1}+b_{2}T}. \end{array}$$

**Figure 2:**
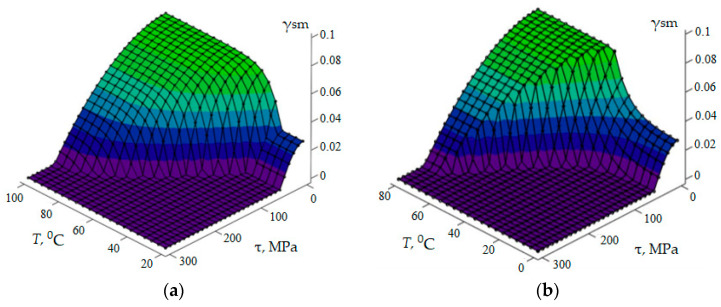
Functional relationship between stress, strain, and temperature of the spring element during reverse (**a**) and forward, (**b**) martensitic transformations.

Graphically, the functional relationship between stress *τ*, strain *γ*, and temperature *T*, as defined by the system of Equation (4) and inequalities (5), is represented by surfaces that illustrate the changes in the thermomechanical characteristics of the actuating element during the forward and reverse phase transformations (Figure 2a,b).

It should be noted that these surfaces are constructed in the coordinates “reactive stress τ—shape memory strain *γ_sm_*—temperature *T*”. In this case, γ*_sm_* = γ*_in_* − γ, and the axis of shear strain *γ_sm_*, corresponding to the shape memory strain, is directed oppositely to the *γ* axis.

The deformation process of the specimen at a constant temperature is described by curves obtained by intersecting surfaces *f* (*τ,γ,T*) = 0 with a plane *T* = const, parallel to plane *τ–γ*. According to Equation (20), during loading, martensitic transformation occurs, the phenomenology of which is described by the second equation in system (4), whereas during unloading, the reverse martensitic transformation takes place, described by the third equation in system (4).

For the given loading and unloading parameters *τ* (*t*), the equations of system (4) can be used to determine the time-dependent behaviour of the martensite volume fraction ξ (*t*) and the strain γ (*t*) at a fixed temperature *T* = *T_i_*.

The stress–strain curve at *T* = *T_i_* will be described by the following equations:

(23) $$\gamma (\tau ,T_{i})={\left(\frac{\tau }{KG(\xi )}\right)}^{\frac{1}{m}}+\gamma_{\mathrm{rev}}(\xi )+\gamma_{\mathrm{res}},\;\xi =f(\tau ,T_{i}).$$

## 4. Results and Discussion

**Figure 3:**
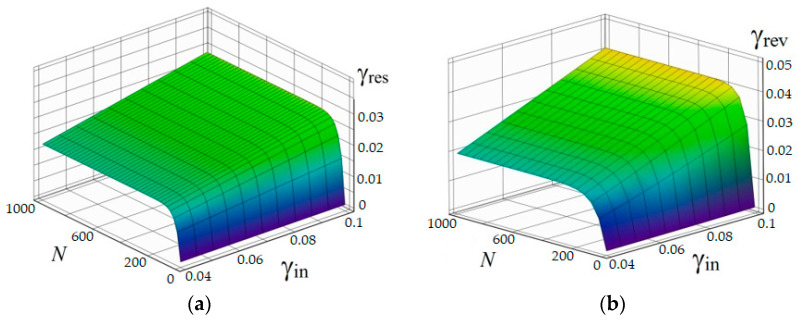
Changing total thermosensitive elements strain during thermomechanical cycling: (**a**) the residual strain, (**b**) reversible strain.

Surfaces $\gamma_{res}=\varphi (N,\gamma_{in})$ and $\gamma_{rev}^{\max}=\varphi^{\prime }(N,\gamma_{in})$ are presented in Figure 3a,b and have standard deviation from the experimental data less than 2%. The study results of thermoelectric processes in the thermosensitive elements can be found in [17].

The values of the constants *C_i_*, *D_i_*, *c_i_*, *d_i_* determined from the experimental data for spring VSP-1 alloy thermosensitive elements are listed in Table 1.

**Figure 4:**
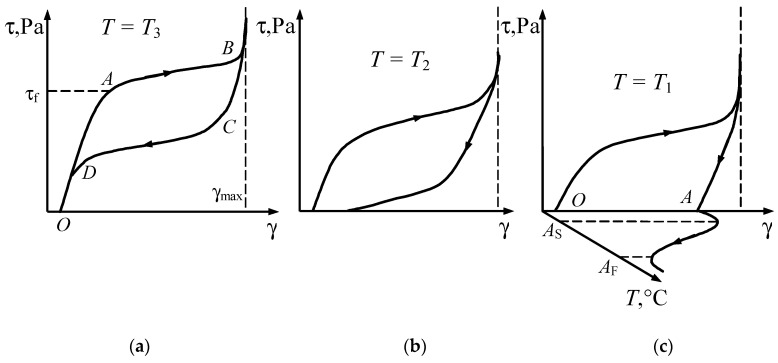
Stress–strain curves at different temperatures *T*_3_ (**a**), *T*_2_ (**b**), and *T*_1_ (**c**).

Figure 4a–c shows the stress–strain curves for the temperatures *T*_1_, *T*_2_, and *T*_3_, which correspond to the temperatures shown in Figure 1.

At temperature *T*_3_ > *A_F_*, from the onset of loading up to point *A*, where the forward martensitic transformation begins, the deformation of the initial phase occurs (Figure 4a). Point *A* corresponds to the phase yield limit of the material. Between points *A* and *B*, the transformation process takes place; in this interval, the microstructure of the material consists of a mixture of martensite and “austenite” phases, and the material exhibits inelastic behaviour.

In order to maximise the working stroke of the actuating element and minimise residual strains, the geometric parameters of the springs should be selected such that the shear strain at point *B* reaches the ultimate phase plasticity strain γ*_ul_* and corresponds to spring compression up to the coil contact. This also contributes to minimising the size of the actuator assembly and prevents exceeding γ*_ul_* under possible overload conditions. If this condition is met, the phase transformation is completed at point *B*, and further loading does not result in additional strain. Upon subsequent unloading, elastic unloading occurs first, up to point *C* (determined by the unloading law [28]), at which point the reverse transformation begins and proceeds along segment *CD*. This is followed by elastic unloading of the “austenite”. At temperature *T*_3_, the “inelastic” strain generated during loading is fully recovered upon unloading, i.e., the material exhibits pseudoelastic behaviour. This property of shape memory alloys makes it possible to manufacture springs with a nonlinear characteristic, whose strain magnitude is nearly an order of magnitude greater than that of comparable springs made from conventional materials.

At temperature *T*_1_ within the range *M_S_* < *T*_1_ < *A_S_* (see Figure 1), during unloading, the stress does not enter the reverse martensitic transformation band. Therefore, the curve τ (γ) ends with elastic unloading of martensite (Figure 4c). The induced strain *OA* is recovered only when the specimen is heated to a temperature above *A_F_*, since in this case the representative point on the phase diagram (Figure 1) moves to the right along the abscissa and crosses the austenitic transformation band. The heating process is shown in plane γ-*T* (Figure 4c). At temperature *T*_1_, the shape memory effect is observed.

At temperature *T*_2_ within the range *A_S_* < *T*_2_ < *A_F_*, only partial austenitic transformation occurs during unloading. The residual strain (Figure 4b) is also recovered upon heating of the spring material. In this case, the material behaviour corresponds to an intermediate state between pseudo elasticity and the shape memory effect.

**Figure 5:**
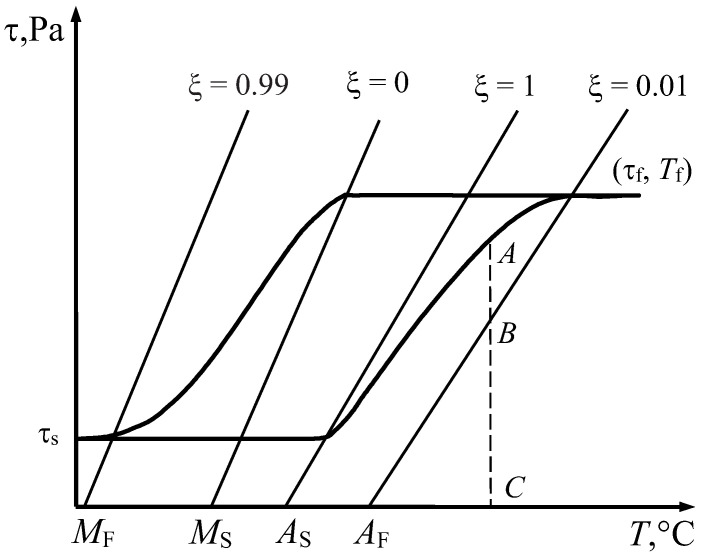
Processes of stress generation and relaxation in phase transformation bands.

The processes of reactive stress generation and relaxation during thermal cycling within the reverse and forward transformation intervals under constant strain conditions are described by curves obtained by intersecting the surfaces *f* (τ,γ,*T*) = 0 for the reverse and forward transformations, respectively, with the plane γ = γ*_i_* = const. From the first equation of system (4), at γ = γ*_i_*, the stress τ (*T*) is determined by assigning temperature values *T*. The second and third equations of the system are then used to determine the value of the internal variable within the phase transformation band (see Figure 5).

As seen in Figure 5, the reverse martensitic transformation is completed upon reaching the temperature *T_f_*. At this point, the reactive stress equals τ*_f_*. If the constraint on strain is then removed, elastic shape recovery will occur (Figure 4c).

It should be noted that if heating of the specimen is stopped at a temperature below *T_f_*, the reverse martensitic transformation halts at point *A* within the reverse transformation band (Figure 5), and the completion of the transformation proceeds between points *A* and *B* during subsequent unloading and strain recovery processes.

The adequacy of the proposed phenomenological model was evaluated by comparing the results of theoretical calculations based on systems of Equations (4) and (5) with experimental data on the thermomechanical characteristics of spring elements made from titanium–nickelide alloys (TN-1K and VSP-1) [14,15,16,17]. The results of investigations into the thermoforce parameters of SMA-based springs have been described in detail in [14,15,16,17].

The values of the shear moduli in the martensitic and parent phases *G_M_* and *G_A_*, the characteristic transformation temperatures, the total residual strain γ*_res_*, the maximum reversible strain $\gamma_{rev}^{max}$, as well as the values of the material parameters *K*, *m*, *a_M_*, *b_M_*, *a_A_* and *b_A_*, determined from experimental data, are presented in Table 2.

**Figure 6:**
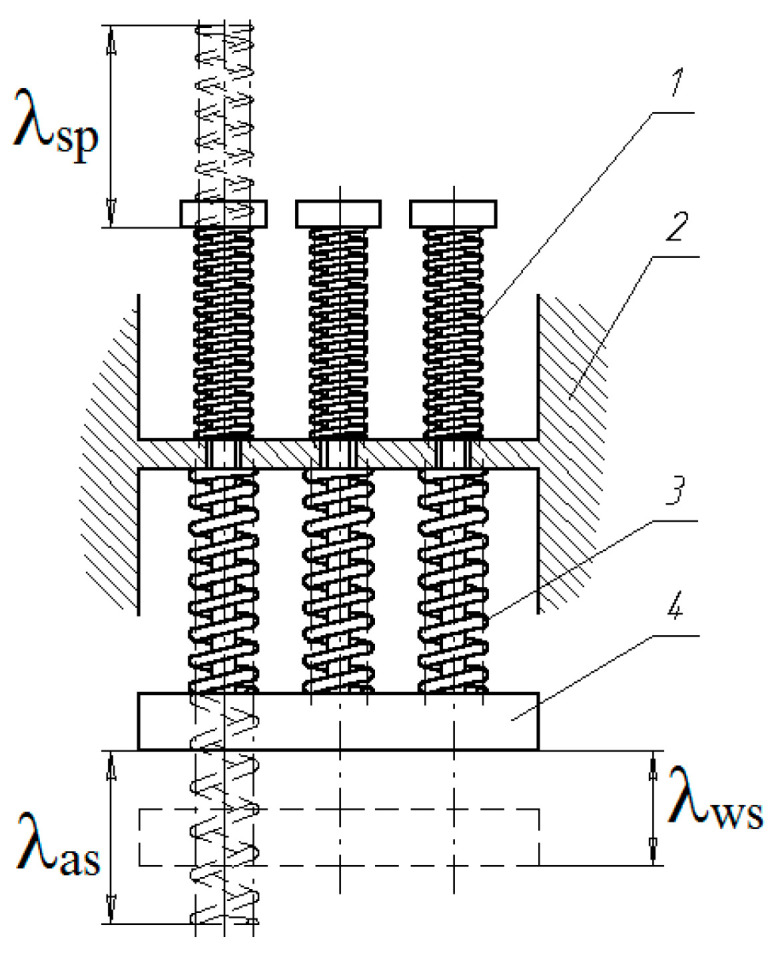
Schematic diagram of the temperature control actuator: 1—return spring, 2—stationary housing, 3—spring with SME, 4—movable link.

The studied springs made of a shape memory alloy were used as thermosensitive elements (TSE) in the actuator of a temperature control regulator [15,16]. An actuator mechanism of the regulator was manufactured, consisting of “*i*” TSEs and “*j*” return springs, with the schematic diagram shown in Figure 6. A significant influence on the functionality of such an actuator is exerted by the constant nature of interaction between the counterbody and the force element. The movable link 4 of the device, connected to the working body, automatically responds to temperature changes in the controlled medium, moving by the value of the working stroke *λ*_ws_ under the action of spring elements 3 with SME and return springs 1, relative to the stationary housing 2. The assembly deformation of the SME springs is equal to *λ*_as_, and that of the return springs is *λ*_sp_.

The calculation of the thermosensitive actuator mechanism of the temperature regulator was carried out based on the proposed relationships (4) and (5). As a result of the design of the thermomechanical unit and its subsequent fabrication, the following characteristics of the force and return springs were obtained:

1. **Springs with SME:** material—TN-1K; number of coils: *n* = 12; free length of the spring *l*_0_ = 85 ± 1 mm; coil pitch *s* = 7 mm (after heat treatment); mean coil diameter *D*_cp_ = 6.2 ± 0.1 mm; wire diameter *d* = 2 mm. After stabilising thermomechanical cycling (1000 cycles), the residual deformation of the springs was *λ*_res_ = 18.3 ÷ 20.4 mm; reversible shape memory deformation *λ*_rev_ = 24.0 ÷ 26.1 mm; shape recovery ratio ~ 100%;
2. **Return Springs:** material—wire $\frac{\text{A}-1-\Pi -1\;\Gamma \text{OCT}\;9389-75}{70\;\Gamma \text{OCT}\;14959-79}$; spring stiffness *c* = 0.869 N/mm; number of active coils *n* = 38.5; total number of coils *n*_1_ = $40.5_{-0.7}^{+0.4}$; outer diameter of the spring *D* = 7.5 ± 0.3 mm; free length of the spring *l*_0_ = $96_{-1.0}^{+3.5}$; working stroke *h* = 23 mm.

**Figure 7:**
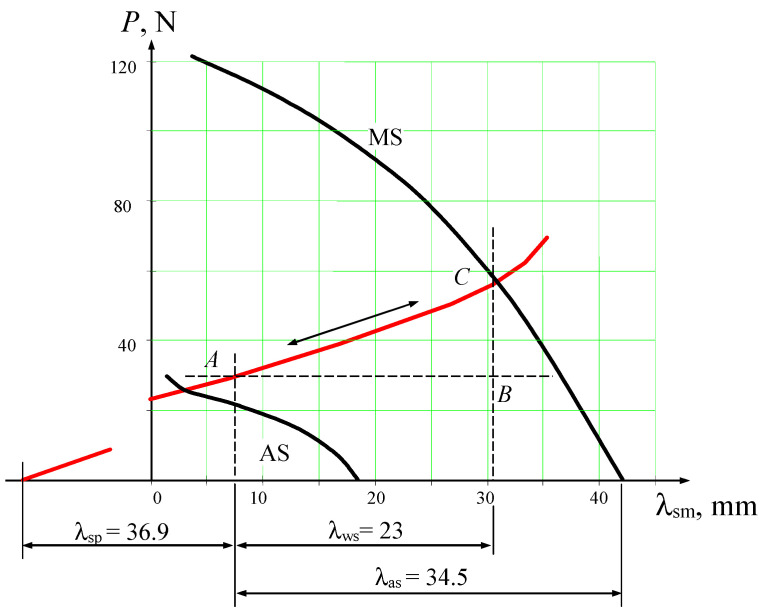
Deformation–force diagram of the temperature control actuator.

The deformation–force diagram, which defines the relationship between the forces generated by the thermoactuator and the displacements of its output link, is shown in Figure 7. It was obtained by projecting the surfaces *f* (*P*,*λ*,*T*), which describe the relationship among force (*P*), deformation (*λ*), and temperature (*T*) parameters of the TSEs during forward and reverse martensitic transformations, and the plane representing the behaviour of the return springs onto a plane perpendicular to the temperature axis *T*.

To determine the output force and the position of the working element depending on the operating temperature, it is necessary to construct intersection curves of the surfaces *f* (*P*,*λ*,*T*) with the deformation plane of the counterbody, on planes perpendicular to the displacement axis *λ*_sm_ and the force axis *P* (lines *CB* and *AB* in Figure 7, respectively).

The forces developed by the given thermoactuator are entirely used to overcome frictional forces in all movable joints during the movement of the working element.

The time constant (response speed) is determined by the rate of temperature change in the thermosensitive elements. Since these elements have small geometric dimensions and a relatively large heat exchange surface area with the controlled medium, the time constant is several seconds (1–2 s), and the thermal regulator as a whole exhibits low inertia.

Based on the conducted experiments [14,15,16,17], it can be concluded that the proposed theory demonstrates satisfactory agreement with the actual stress–strain-temperature (τ-γ-T) relationships. The discrepancy Δτ = τ (γ, T) − τe (where τe is the stress determined from experimental data) remains within the experimental error margin (6.5% of τe). The root-mean-square deviation of Δτ does not exceed 5%. With an appropriate modification of the first constitutive equation in system (4), the described phenomenological model can be employed to determine the relationship between strain, force, and temperature parameters of actuating elements subjected to axial and bending deformations.

## 5. Conclusions

The research conducted confirms the suitability of a phenomenological approach for modelling the thermomechanical behaviour of shape memory alloy (SMA) spring elements. Based on the analysis and comparison with experimental data, the following conclusions can be drawn:

1. Macroscopic phenomenological models based on integral material characteristics are appropriate for simulating the functional parameters of thermosensitive SMA elements used in control devices and cyclic actuators. These material parameters can be reliably obtained under laboratory conditions by manufacturers or design organisations.
2. The behaviour of cylindrical SMA springs under thermomechanical loading can be effectively analysed using established theories of helical spring deformation, including large displacement mechanics and stress analysis beyond the proportional limit, provided the influence of temperature on stress–strain relations is considered.
3. Applying the internal variable concept from continuum mechanics allows for a physically grounded description of the relationship between mechanical response and the kinetics of phase transformation. This enables accurate predictions across the full operational temperature range of SMA-based actuators in technical systems.

## Figures and Tables

**Table 1:** The material parameters of the spring thermosensitive elements (alloy VSP-1).

*u*_i_(γ*_in_*)		*u*_0_	*u*_1_	*u*_2_	*ν*_i_(γ*_in_*)		*ν*_0_	*ν*_1_	*ν*_2_
γ*_res_*	*C_i_*	0.226	−0.226	0.049	$\gamma_{rev}^{max}$	*c_i_*	0.43	−0.446	−0.302
	*D_i_*	5.466 × 10^−3^	−5.314 × 10^−3^	−0.035		*d_i_*	−2.188 × 10^−3^	2.588 × 10^−3^	−0.016

**Table 2:** Material parameters of the springs (TN-1K alloy) *.

*G_A_*, [MPa]	*G_M_*, [MPa]	*M_F_*, [°C]	*M_S_*, [°C]	*A_S_*, [°C]	*A_F_*, [°C]	*γ_res_*	$\gamma_{rev}^{max}$	*a_A_*, [1/MPa]	*a_M_*, [1/MPa]	*b_A_*, [MPa/°C]	*b_M_*, [MPa/°C]	*K*	*m*
28 × 10^3^	9 × 10^3^	1.9	31.7	40.9	65.0	0.029	0.041	0.029	−0.019	6.723	7.926	0.037	0.533

* The material parameters were determined after stabilising thermomechanical cycling: *N* = 1000, *T_max_* = 100 °C, *T_min_* = 0 °C; γ*_in_* = 0.0964.

## Data Availability

The original contributions presented in the study are included in the article, further inquiries can be directed to the corresponding author.
